# Generation of Recombinant Antibodies against the beta-(1,6)-Branched beta-(1,3)-D-Glucan Schizophyllan from Immunized Mice via Phage Display

**DOI:** 10.1155/2017/8791359

**Published:** 2017-05-23

**Authors:** Jörn Josewski, Sabine Buchmeier, André Frenzel, Philip Tinnefeld, Stefan Dübel, Udo Rau

**Affiliations:** ^1^Department for Biotechnology, Institute of Biochemistry, Biotechnology and Bioinformatics, Technische Universität Braunschweig, Spielmannstraße 17, 38106 Braunschweig, Germany; ^2^Institute for Physical and Theoretical Chemistry, Technische Universität Braunschweig, Rebenring 56, 38106 Braunschweig, Germany; ^3^YUMAB GmbH, Rebenring 33, 38106 Braunschweig, Germany

## Abstract

beta-(1,6)-Branched beta-(1,3)-D-glucans like schizophyllan from the basidiomycete* Schizophyllum commune* excite various immunostimulatory effects and have been clinically tested as adjuvants. Some of the glucans are also applicable in food or petrol industry due to their viscosity and temperature stability in aqueous solution. Antibodies against these glucans could be used as tool for analysis of glucan preparations or for further research of its bioactivity. Therefore, an immune phage display library was constructed from mice immunized with schizophyllan. Three recombinant monoclonal antibodies were isolated from this library by affinity selection (panning) on schizophyllan. The half-maximal effective concentration (EC50) values for those antibodies varied between 16.4 ng mL^−1^ and 21.3 ng mL^−1^. The clones showed binding specificity not only for schizophyllan but also for other beta-(1,6)-branched beta-(1,3)-D-glucans of similar macromolecular structure. Denaturation of the secondary structure led to a reduced antibody binding, indicating an epitope requiring the correct conformation of the triple helical structure of the glucans.

## 1. Introduction

A variety of fungal beta-(1,3)-D-glucans are described as substances with antitumoral and immunomodulating activities [1]. One example is schizophyllan (SCH) that is produced as extracellular polysaccharide by the basidiomycete* Schizophyllum commune* [2–5]. Its bioactivity is based on the enhancement of cell-mediated immune response with stimulation of T lymphocytes and macrophages and improving cytokine production [6–9].

The primary molecular structure of SCH consists of a beta-(1,3)-D-glucan main chain with single beta-(1,6)-linked glucose molecules at approximately every third glucose monomer of the backbone [10, 11]. In aqueous solution it forms the secondary structure of a triplex composed of three twisted chains stabilized by hydrogen bonds with single beta-(1,6)-linked glucose residues protruding outside the helix backbone [12–15]. Differences in branching grade and molecular weight are considered to be responsible for variations in bioactivity [16–18]. In addition to its bioactivity, SCH also has physical properties favorable for a use in food and oil industry. Aqueous solutions of SCH are viscous and show pseudoplastic flow behavior [19]. Furthermore, they are stable at high temperature and in a broad pH range, starting to denature at pH > 12 or >135°C [15, 20, 21]. Since SCH can be used for many applications, the generation of antibodies would be of great value because they could be used for quantitative trace analysis or for the investigation of the role of particular conformations for glucan bioactivity.

The objective of this study was the generation of recombinant monoclonal antibodies (rAbs) against the beta-D-glucan schizophyllan. Therefore, we constructed an antibody phage display library from the lymphocytes of three mice which had been immunized with proteinase K treated SCH (SCH-PK). After panning of this library for SCH-PK binding antibody phage, we were able to derive three rAbs specificity binding beta-(1,6)-branched beta-(1,3)-D-glucans with the same secondary structure as SCH.

## 2. Materials and Methods

### 2.1. Chemicals

All chemicals were purchased from Sigma Aldrich if not mentioned otherwise.

### 2.2. Preparation of beta-(1,6)-Branched beta-(1,3)-D-Glucans

Following beta-(1,6)-branched beta-(1,3)-D-glucans were prepared from biomass-free and stabilized (5 g L^−1^ formic acid) culture supernatants: SCH (*Schizophyllum commune *ATCC 38548), scleroglucan (*Sclerotium rolfsii *ATCC 15205), cinerean (*Botrytis cinerea *LU 14548), and fructican (*Monilinia fructigena *ATCC 24976) [4, 22–24]. The supernatants were diluted to 1.0 g L^−1^ glucan (volume 100 mL) with water and adjusted to pH 7.5 with NaOH. In the next step the glucan solutions were purified by diafiltration with water (Vivaflow 50 R, MWCO 100,000, Sartorius, Göttingen, Germany) until the conductance of the retentate dropped to 0 *µ*S cm^−1^. The glucan solution was then reduced to 100 mL and sterilized at 120°C for 20 minutes. Quantification was performed by mixing of 5 mL glucan solution with 15 mL 2-propanol and incubation overnight at 4°C. The precipitated glucan was weighed after isolation by centrifugation with 13,000 ×g at 4°C as well as drying for 48 h at 50°C under reduced pressure.

In addition to this schizophyllan preparation, commercially acquired samples of schizophyllan from Contipro Biotech (Dolní Dobrouč, Czech Republik) and Actigum™ CS 11 (scleroglucan) purchased from Degussa Construction Polymers GmbH (Trostberg, Germany) were used.

### 2.3. Preparation of Proteinase K Treated Glucans

SCH additionally treated with proteinase K (SCH-PK) was used as antigen for the isolation of antibodies. For its preparation, biomass-free culture supernatant of* S. commune* was diluted to 1.0 g L^−1^ glucan in 100 mL with water and adjusted to pH 7.5 with NaOH. 2 mL of 0.5 mol L^−1^ CaCl_2_ in 1 mol L^−1^ Tris-HCl (pH 7.4) and 1 mL of 10% (w/v) sodium dodecyl sulfate were added and the solution was incubated at 80°C for 4 hours. After cooling to 40°C, 10 mg of proteinase K was solved and the solution incubated for 24 h at 40°C under slight shaking. The proteinase K treatment was stopped by incubation at 80°C for 4 hours. The solution was diluted to 1 L with water, cleared by centrifugation with 13,000 ×g for 1 h at 16°C and then processed as described above. The same procedure was applied for culture supernatant of* S. rolfsii.*

### 2.4. Immunization of Mice and Library Construction

Three mice (BALB/c, female, 7 weeks old) were immunized with SCH-PK by intraperitoneal injection. Three injections were applied in two-week intervals. Each injection consisted of 25 *µ*g of SCH-PK and 50 *µ*L of the adjuvant Magic™ Mouse (Creative Biomart, New York, USA). Four weeks after the third injection, three additional injections with 50 *µ*g of SCH-PK and 100 *µ*L of PBS (phosphate buffered saline) were applied over a period of three days to boost the B cell proliferation. Two days later, the mice were sacrificed. Their spleens were isolated and homogenized in TRIzol® LS Reagent (Life technologies, Carlsbad, USA).

A volume equal to 1 × 10^7^ of homogenized leucocytes was used to isolate the RNA with the Direct-zol MiniPrep kit (Zymo Research, Irvine, USA). The isolation of the rearranged genes for the antibody domains *V*_*L*_ (variable light) and *V*_*H*_ (variable heavy) and their cloning into the phage display vector pHAL30 was carried out as described [25, 26]. The derived *V*_*L*_ sublibraries for lambda (V-LAMBDA) and kappa (V-KAPPA) of each mouse were kept separately. The six sublibraries were packaged with hyperphage [27].

### 2.5. Ethics Statement

The experimental protocols were carried out in accordance with the Directive 2010/63/EU of the European Parliament and the Council of the European Union of 22 September 2010 and all procedures were approved by guidelines from the Animal Committee on Ethics in the Care and Use of Laboratory Animals of TU Braunschweig, Germany (Az §5 (02.05) TschB TU BS Az:33.42502-14-005/08).

### 2.6. Immobilization of Schizophyllan onto Multiwell Plates

For the antibody selection and ELISA experiments, schizophyllan was immobilized to Carbo-BIND multiwell plates (Corning, Corning, USA). The wells were filled with 100 *µ*L of 10 *µ*g mL^−1^ SCH-PK in 10 mmol L^−1^ acetate buffer (pH 5.4) and the sealed plates were incubated at 37°C overnight. The following day the wells were washed 10 times with water in an ELISA washer (HydroFlex™, Tecan, Männedorf, Switzerland) and filled for storage with 300 *µ*L PBS (8.0 g L^−1^ NaCl, 0.2 g L^−1^ KCl, 1.44 g L^−1^ Na_2_HPO_3_*∗*4H_2_O, 0.24 g L^−1^ KH_2_PO_4_, pH 7.4) containing NaN_3_ (0,2 g L^−1^) until use.

### 2.7. Selection of Recombinant Antibodies against SCH by Antibody Phage Display

The rAbs were selected by panning as described [28] from the pooled immune libraries with V-LAMBDA (3 × 10^10^ colony forming units, cfu) and V-KAPPA (3 × 10^10^ cfu) which had been preincubated for 1 h in blank wells of a Carbo-BIND multiwell plate. Plasmid preparations (NucleoSpin® Plasmid EasyPure, Macherey-Nagel, Düren, Germany) were derived from the* E. coli* clones which contained a positive tested rAB and sent to Seqlab Sequence Laboratories GmbH (Göttingen, Germany) for sequencing. The sequences were compared with mouse germline sequences via the online sequence analysis tool IMGT/V-QUEST from the International ImMunoGeneTics information system® (IMGT®, http://www.imgt.org/) [29, 30].

### 2.8. Production and Purification of Isolated Antibodies as scFv-Fc

For further characterization of the isolated rAbs, the DNA encoding the scFv (single chain Fragment variable) was subcloned into pCSE2.6-mIgG2c-Fc-XP via NcoI and NotI (New England Biolabs, Ipswich, USA). The resulting scFv-Fc fusions (scFv fused with a murine Fc part) were produced in HEK239-6E cells (National Research Council, Biotechnological Research Institute, Montreal, Canada) as described [31]. Briefly, cells were cultivated in chemically defined medium FreeStyle™ F17 (Gibco™, Thermo Fisher Scientific, Waltham, USA) added by 1 g L^−1^ Pluronic® F-68 (AppliChem, Darmstadt, Germany), 4 mmol L^−1^ glutamine (PAA Laboratories GmbH, Cölbe, Germany), and 25 mg L^−1^ G418 (PAA Laboratories GmbH, Cölbe, Germany) at 37°C, 110 rpm as well as 5% CO_2_ in the atmosphere (Minitron, Infors, Bottmingen, Switzerland). For transfection, 25 *µ*g of expression vector was used for 25 mL culture (about 1.5 × 10^6^ cells mL^−1^) in a 125 mL Erlenmeyer shake flask. 48 h after transfection the culture was fed with 25 mL fresh media and 1.25 mL of 20% (w/v) tryptone N1 (Organotechnie S.A.S, La Courneuve, France). The secreted scFv-Fc was purified from the supernatant by affinity chromatography on a UNOsphere SUPrA™ column (Biorad, Hercules, USA) and a Bio-Scale™ Mini Bio-Gel®P-6 Desalting cartridge (Biorad, Hercules, USA) in an automated Profinia™ system (BioRad, Hercules, USA). The concentrations of the preparations were calculated by their absorbance at 280 nm (NanoDrop 2000, Thermo Fisher Scientific, Waltham, USA).

### 2.9. Titration ELISA

The half-maximal effective concentration (EC50) values of the produced scFv-Fc were determined by titration ELISA (enzyme-linked immunosorbent assay) using serial dilutions of the scFv-Fc in blocking solution (2% (w/v) milk powder and 0.05% (w/v) Tween® 20 in PBS). ELISA was performed with SCH-PK loaded Carbo-BIND multiwell plates (100 *µ*L well^−1^, 10 *µ*g mL^−1^). The plate was blocked for 1 h at room temperature. The blocking solution was discarded and 100 *µ*L of the scFv-Fc (in blocking solution) was filled into the wells. After 1 h incubation, the plate was washed for 3 times with PBST (0.05% (w/v) Tween 20 in PBS). Afterwards, 100 *µ*L well^−1^ peroxidase-conjugated goat anti-murine Fc antibody (A0168, 1 : 40,000 in blocking solution) was added and incubated for an additional 1 h. The plate was then washed again for 3 times with PBST. The color reaction was obtained by adding 100 *µ*L well^−1^ TMB solution (20 volumes TMB A (30 mmol L^−1^ potassium citrate, 0.5 mol L^−1^ citric acid, pH 4.1) and 1 volume TMB B (10 mmol L^−1^ 3,3′,5,5′-tetramethylbenzidine, 10% (v/v) acetone, 90% (v/v) ethanol, 0.3% (v/v) H_2_O_2_)). The reaction was stopped after 10 min by addition of 100 *µ*L well^−1^ of 0.5 mol L^−1^ H_2_SO_4_. The resulting absorption was measured at 450 nm (reference wavelength 620 nm).

### 2.10. Competitive ELISA

Antigen specificity was evaluated via competition of scFv-Fc binding to immobilized SCH-PK by various soluble saccharides. Each scFv-Fc was used in the concentration that resulted in 40% of the saturation signal in the titration ELISA. They were preincubated in serial dilutions of competitors (3.2 ng mL^−1^ to 1 mg mL^−1^, blocking solution as solvent) for 1 h before they were transferred to the SCH-PK loaded Carbo-BIND plate. The further procedure was done as described for titration ELISA. The following substances were used as competitors: SCH-PK, SCH, scleroglucan* (S. rolfsii)*, cinerean, fructican, scleroglucan of* Sclerotium glucanicum* (DSM 2159), SCH from Contipro Biotech, Actigum CS 11 (scleroglucan), laminarin, dextran, xanthan, laminarihexaose (Megazyme, Wicklow, Ireland), beta-(1,6)-D-gentiobiose, yeast extract (Ohly, Hamburg, Germany), and glucose. Furthermore, NaOH treated SCH-PK was used, due to its changed secondary structure (triplex denatured to single chains, partial formation of cyclic structures). It was derived by adding NaOH until a pH of 13.8 had been reached and it was neutralized with HCl before use. Solutions of denatured SCH-PK and SCH were also used. Both were prepared by overnight incubation of SCH-PK and SCH with endoglucanase from* Penicillium funiculosum* (kindly provided by Erbslöh, Geisenheim, Germany) at 50°C. The endoglucanase was inactivated by heating up to 80°C for 20 minutes.

All investigations were carried out at least in three independent experiments.

## 3. Results and Discussion

### 3.1. Selection and Identification of Recombinant Antibodies against Proteinase K Treated Schizophyllan

The goal of this study was the generation of a rAb that binds to the beta-(1,6)-branched beta-(1,3)-D-glucan schizophyllan of* Schizophyllum commune*. We constructed a library from three immunized mice to increase the possibility of isolating the desired antibody. Since we wanted to derive an antibody which binds to the glucan itself, rather to possible residual protein contaminants, we used proteinase K treated SCH for immunization. The proteinase K treatment should eliminate proteins or other polypeptides that could be associated with the glucan. From each mouse, two sublibraries were derived, V-LAMBDA and V-KAPPA, which were pooled and used for the antibody selection on SCH-PK loaded Carbo-BIND multiwell plates. The surface of Carbo-BIND plates is functionalized with hydrazide-groups. Hydrazides react selectively with aldehyde groups under acidic conditions which allowed the covalent binding of the reducing end from SCH. Preliminary experiments showed that multiwell plates with other surface properties did not work and sometimes led to the generation of antibodies against adsorbed protein impurities (data not shown).

After three rounds of panning, 92 of the enriched antibody clones from each library were selected and the respective soluble scFv-fragments were tested by ELISA for binding to SCH-PK. 60 clones of the V-KAPPA library showed specific binding; 10 of those clones with the highest signal and highest difference to the signal of the negative control were sequenced. Three individual antibodies (JoJ48C11, JoJ48F1, and JoJ49D10) with high sequence similarities were identified. The heavy chain of each recombinant antibody contains the variable gene IGHV1-7*∗*01, the diversity gene IGHD1-1*∗*02, and the joining gene IGHJ2*∗*01. The variable gene of the light chain for each antibody is IGKV4-59*∗*01. For the antibodies JoJ48C11 and JoJ49D10 the joining gene is IGKJ5*∗*01. IGKJ2*∗*07 was assigned to JoJ48F1.

### 3.2. Binding Strength of the Isolated Antibodies as scFv-Fc

The three isolated rAbs were produced as scFv-Fc consisting of the scFv fused to a murine IgG2c Fc part. The scFv-Fc format is a bivalent antibody comparable to a full length IgG [31].

**Figure 1:**
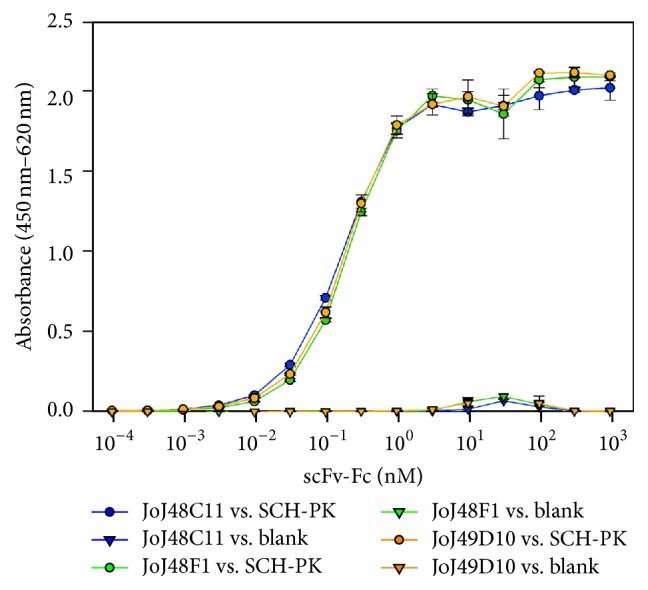
Titration ELISA for analysis of the binding of the isolated antibodies in bivalent form (scFv-Fc). Dilution series of the antibodies from 0.095 pM to 945 nM were applied to SCH-PK coated or uncoated Carbo-BIND™ plates.

The rAbs were analyzed as scFv-Fc by titration ELISA. Serial dilutions from 0.095 pM to 945 nM antibody were applied on SCH-PK loaded and blank Carbo-BIND multiwell plates. The resulting data (Figure 1) showed almost identical sigmoidal increase of the absorbance signal with increasing scFv-Fc concentration in a half-logarithm plot for all three antibodies. The half-maximal effective concentration (EC50) was derived from a regression of absorbance signal against the logarithm of antibody concentration by a sigmoidal function (3 parameters). The EC50 correlates with the affinity of the rAbs to SCH-PK [32]. The EC50 values are 155 ± 4 pM, 201 ± 7 pM and 189 ± 9 pM for JoJ48C11, JoJ48F1, and JoJ49D10, respectively.

### 3.3. Antibody Specificity

**Figure 2:**
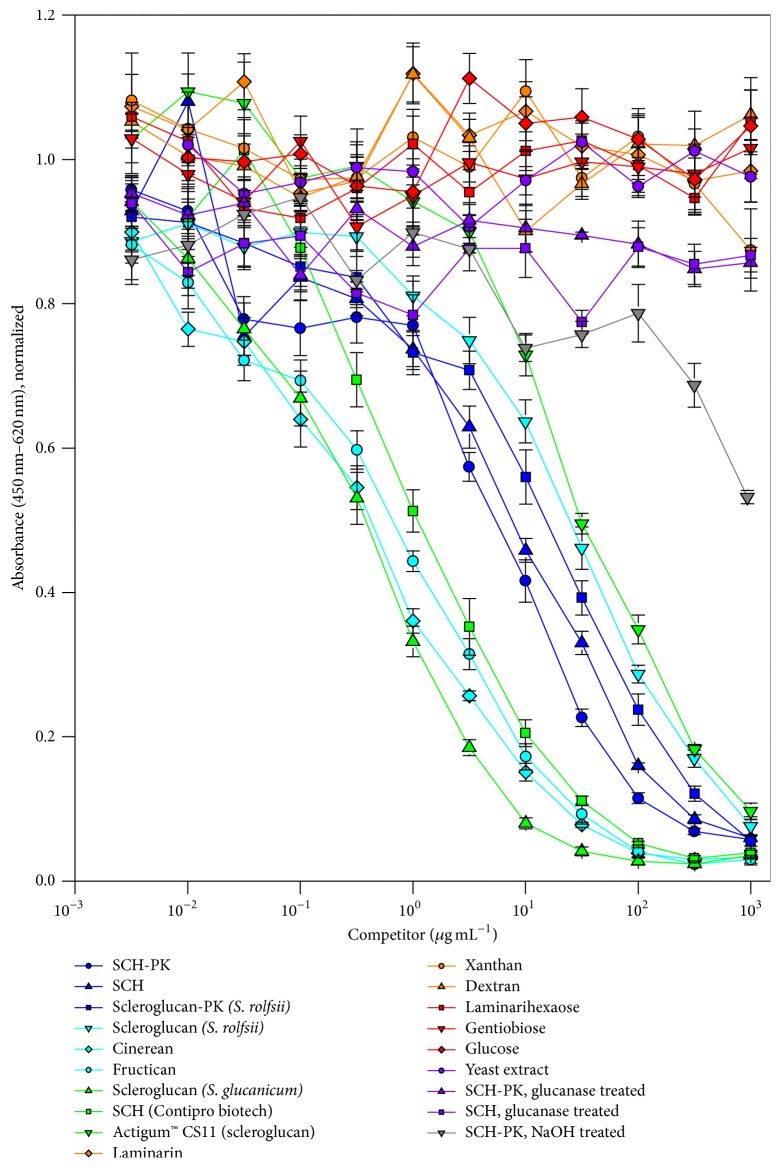
Competitive ELISA of antibody JoJ49D10 for the characterization of antigen specificity. JoJ49D10 (scFv-Fc) was preincubated in dilutions of different carbohydrates or yeast extract ranging from 10 ng mL^−1^ to 1 mg mL^−1^ before being added to SCH-PK loaded Carbo-BIND multiwell plates. The absorbance signals were normalized by division with the absorbance of respective samples without competitor.

Analysis for the specificity in antigen recognition was performed by a competitive approach. The rAbs were incubated in solutions of different saccharides or yeast extract before they were added to SCH-PK loaded Carbo-BIND plates. For illustration, only the data of JoJ49D10 are presented in Figure 2 because the results of JoJ48C11 and JoJ48F1 were very similar.

The expected decrease of signal with increasing concentrations of SCH-PK in solution was observed for each rAb. Furthermore, a decrease of signal was also found, as expected, for nontreated SCH and the other beta-(1,6)-branched beta-(1,3)-D-glucans like scleroglucan of* S. rolfsii*, cinerean, fructican, scleroglucan of* S. glucanicum*, proteinase K treated scleroglucan, SCH (Contipro Biotech), and Actigum CS 11 (scleroglucan from Degussa Construction Polymers). The scleroglucans and cinerean have a high similarity to SCH. They possess the same primary molecular structure, consisting of beta-(1,6)-linked D-glucose at every third glucose of the beta-(1,3)-D-glucan main chain. Additionally, they form also a triple helix as secondary structure [22, 23]. Fructican has a slightly different primary structure with beta-(1,6)-bound D-glucose alternating at every second or third glucose of the main chain [18, 24]. Its secondary structure is considered to be a triplex as well. The recognition of those glucans reveals a specificity of the rAbs not just for SCH but also for other high molecular beta-(1,6)-branched beta-(1,3)-D-glucans forming a triple helix.

Only a slight signal decrease at higher concentrations of NaOH treated SCH-PK was observed for each rAb. Due to the NaOH treatment of SCH-PK, the glucan possesses a changed macromolecular structure. The triple helix denatures into single chains when the pH exceeds 13 [21]. Neutralization of those SCH solutions leads to minimal renaturation to the triple helical structure, while random coiled structures are primarily formed [33]. This observation indicates that the native triple helical structure is an important factor for the recognition of the beta-(1,6)-branched beta-(1,3)-D-glucan molecule by the selected antibodies, since the primary structure is not changed. The residual interaction could be explained by occasional formation of triple helical stretches inside the random coils and small amounts of renatured SCH triplex [34].

JoJ48C11 and JoJ48F1 showed a slight interaction with laminarin of* Laminaria digitata*. It consists of a beta-(1,3)-D-glucan main chain with an occasional beta-(1,6)-bound D-glucose and exists predominantly as linear molecule with only approximately 5% occurring as triplex [35]. This observation further corroborates the assumption that the triple helical structure is a key factor for rAb recognition.

Xanthan, dextran, laminarihexaose, beta-(1,6)-D-gentiobiose, glucose, yeast extract, and glucanase treated SCH/SCH-PK did not inhibit the binding of any of the three antibodies to SCH-PK. Xanthan consists of a beta-(1,4)-D-glucan backbone with beta-(1,3)-linked beta-D-mannose-(1,4)-beta-D-glucuronic acid-(1,2)-alpha-D-mannose side chain at every second glucose and forms a double helix in aqueous solution [36]. Dextran is an alpha-(1,6)-D-glucan with around 5% branching points at alpha-(1,3)-position to a single glucose or a dimer of glucose [37]. The results with xanthan and dextran indicate that there are no unspecific interactions of the rAbs with high molecular polysaccharides of different glycosidic linkages. D-glucose and laminarihexaose (linear hexamer of beta-(1,3)-bound D-glucose) and beta-(1,6)-D-gentiobiose (dimer of beta-(1,6) bound D-glucose) also did not interfere with the binding of the rAbs to SCH, indicating that D-glucose and the beta-(1,6) or beta-(1,3) linkage of D-glucose alone are not sufficient to provide binding by the rAbs. Yeast extract was the complex ingredient of the medium for the production of SCH. The test assured that the antibodies do not bind to a residual impurity from the media in the used SCH solution. Incubation of SCH with endoglucanase degrades it into D-glucose monomers and oligomers. An interference of the glucanase treated solutions was not observed and indicated that degraded saccharides or other released components, including potential protein impurities, were not recognized by the antibodies.

The experiments showed that the triple helical structure of the glucans is the essential factor for antibody recognition. It is assumed that the spatial arrangement of the beta-(1,6)-linked D-glucose residues is an important part of the antibody binding epitope. The beta-(1,6)-branching ratio of the glucan also affects the reactivity of the antibody binding. Fructican possesses a higher degree of branching points but does not influence the reactivity of the antibodies compared to the different types of schizophyllan and scleroglucan as well as cinerean. Laminarin shows not only a low occurrence of triplex structure but also a lower degree of branching which could be also responsible for the weak antibody binding. This leads to the additional conclusion that besides the triplex the beta-(1,6)-branching degree also influences antibody reactivity.

With the gained datasets the EC50 values of the competing glucans (except for NaOH treated SCH-PK and laminarin) were calculated from the signal reduction and compared as value for the binding strength of the antibodies to the glucans (all data shown in Table 1). In dependence of the rAb, the EC50 values for SCH-PK varied between 7.8 to 25.1 *µ*g mL^−1^. The proteinase K untreated SCH offered jointly increased EC50 of 9.1 to 36.2 *µ*g mL^−1^. The lowest EC50 values of 0.4 to 3.1 *µ*g mL^−1^ and consequently the greatest interference were observed for scleroglucan from* S. glucanicum*. The difference of EC50 values between the glucans can be explained by different molecular size distribution, occurrence of additional macromolecular structures depending on the production strains, and downstream processing. For example, SCH and SCH-PK showed similar EC50 values because both derived from the same production batch and were identically purified except proteinase K treatment. The commercial SCH purchased by Contipro Biotech, produced and purified in unknown procedures, showed lower EC50 data.

In summary, the results suggest that the generated rAbs recognize a conformational epitope requiring an intact triple helical secondary structure of beta-(1,6)-branched beta-(1,3)-D-glucans with an optimal branching degree of one beta-(1,6)-bound D-glucose attached to approximately every three glucose units of the main chain. As a consequence, the rAbs not only are specific for SCH but also bind to similar beta-(1,6)-branched beta-(1,3)-D-glucans with triple helical structure.

Reports about the generation of an antibody against SCH already exist. Tabata et al. reported the generation of an antiserum from immunized rabbits [38]. Other authors described the isolation of a monoclonal antibody against SCH from immunized mice via hybridoma technology [39]. That antibody was also able to bind other beta-(1,6)-branched beta-(1,3)-D-glucans like scleroglucan and even bound to laminaritetraose, a tetramer of beta-(1,3)-bound D-glucose.

Investigations of monoclonal antibodies generated with other beta-(1,3)-D-glucans also exist with binding specificities to more than one glucan [40–43]. Just recently, two antibodies against the extracellular beta-glucan of* Pleurotus ostreatus* were isolated [44, 45]. This glucan exhibits the same molecular structure as SCH. It is assumed as well that these antibodies recognize and bind to a common conformational epitope. The difference to our investigations is that we proved the triple helical arrangement as an essential element for the recognition by the rAbs. Furthermore, it was verified by proteinase and endoglucanase treatment that the rAbs bind to the sugar structure and not to associated proteins or peptides.

## 4. Conclusion

In this report the successful generation of three recombinant glucan antibodies is described. They were selected for binding to the beta-(1,6)-branched beta-(1,3)-D-glucan schizophyllan of* Schizophyllum commune* from an immune library via phage display. In addition to schizophyllan, the antibodies recognize other beta-(1,6)-branched beta-(1,3)-D-glucans that possess a triplex as secondary structure and similar branching degree as schizophyllan. As other helical arrangements and lower order oligomers or monomers were not bound, the antigenic epitope is assumed to be conformational. Therefore, the antibodies can be used as analytical tools for the specific detection of schizophyllan and similar beta-(1,6)-branched beta-(1,3)-D-glucans with triple helical arrangement.

## Figures and Tables

**Table 1:** EC50 values of competitors which are recognized by the recombinant antibodies.

Competitors	EC50 values [*µ*g mL^−1^]		
	JoJ48C11	JoJ48F1	JoJ49D10
SCH-PK	25.1 ± 1.8	24.9 ± 3.7	7.8 ± 1.0
SCH	36.2 ± 4.6	29.4 ± 6.4	9.1 ± 0.6
Scleroglucan *(S. rolfsii)*	54.4 ± 2.0	52.3 ± 5.3	19.9 ± 2.8
Scleroglucan (PK-treated)	83.1 ± 5.1	54.5 ± 13.2	34.0 ± 1.2
Cinerean	4.2 ± 0.2	4.7 ± 0.7	0.5 ± 0.1
Fructican	6.8 ± 0.4	8.8 ± 1.8	1.0 ± 0.2
Scleroglucan *(S. glucanicum)*	3.1 ± 0.2	1.7 ± 0.5	0.4 ± 0.1
SCH (Contipro biotech)	8.2 ± 1.2	7.0 ± 1.2	1.1 ± 0.2
Actigum CS11 (Scleroglucan)	88.5 ± 5.0	74.2 ± 14.4	31.7 ± 1.5
